# Accelerated oxygen-induced retinopathy is a reliable model of ischemia-induced retinal neovascularization

**DOI:** 10.1371/journal.pone.0179759

**Published:** 2017-06-26

**Authors:** Pilar Villacampa, Katja E. Menger, Laura Abelleira, Joana Ribeiro, Yanai Duran, Alexander J. Smith, Robin R. Ali, Ulrich F. Luhmann, James W. B. Bainbridge

**Affiliations:** Division of Genetics, UCL Institute of Ophthalmology, University College London, London, United Kingdom; Children's Hospital Boston, UNITED STATES

## Abstract

Retinal ischemia and pathological angiogenesis cause severe impairment of sight. Oxygen-induced retinopathy (OIR) in young mice is widely used as a model to investigate the underlying pathological mechanisms and develop therapeutic interventions. We compared directly the conventional OIR model (exposure to 75% O_2_ from postnatal day (P) 7 to P12) with an alternative, accelerated version (85% O_2_ from P8 to P11). We found that accelerated OIR induces similar pre-retinal neovascularization but greater retinal vascular regression that recovers more rapidly. The extent of retinal gliosis is similar but neuroretinal function, as measured by electroretinography, is better maintained in the accelerated model. We found no systemic or maternal morbidity in either model. Accelerated OIR offers a safe, reliable and more rapid alternative model in which pre-retinal neovascularization is similar but retinal vascular regression is greater.

## Introduction

Pathological angiogenesis is a common feature of many conditions, including blinding eye diseases and cancer. Oxygen-induced retinopathy (OIR) in young mice, originally developed as model of retinopathy of prematurity [1], is widely used to investigate mechanisms of ischemia-induced neovascularization, and to develop therapeutic interventions. In OIR, exposure of mouse pups to 75% oxygen from postnatal day (P)7 induces the regression of the developing central retinal vasculature [1, 2]. On return to room air at P12, the resulting local ischemia induces both appropriate revascularization of the avascular retina, and the development of aberrant neovascular tufts that extend from the surface of the inner retina into the vitreous. These neovascular tufts, which can be quantified in flat-mounted retinas or histological sections, reach their greatest extent at P17 before regressing spontaneously [2]. An alternative, accelerated model of OIR, in which mice are exposed to 85% oxygen from P11 to P16 has been described [3, 4]. We sought to determine the value of this accelerated version by performing a direct comparison with the conventional model. We compared the extents of retinal vascular regression, revascularization and pre-retinal neovascularization. We also measured retinal gliosis, neuroretinal function and maternal hyperoxia-induced lung injury.

## Materials and methods

### Ethics statement

All animal studies were carried out under the Animals (Scientific Procedures) Act 1986 under a project license PPL 70/8120 issued by the UK Government Home Office and conducted in accordance with protocols approved by the Animal Welfare and Ethics Committee of the UCL Institute of Ophthalmology. All animals were killed by trained personnel using cervical dislocation (approved under Schedule 1 as a method of humane killing). All efforts were made to minimize their number and distress.

### Animals

C57Bl/6J (Harlan, UK) mice were maintained in the animal facility at University College London. Mothers and pups of both sexes were kept on a standard 12/12 hour light/dark cycle and at the same light levels throughout and used at the ages specified for each protocol.

### OIR protocols

We exposed litters of C57Bl6 mice (n = 6–14) to 85% oxygen from P8 to P11 (the accelerated model), or to 75% oxygen from P7 to P12 (the conventional model). Numbers of mice used for each experiment were calculated following previous experiences. No more than 6 pups for litter were placed in the chamber, in order to ensure sufficient feeding and normal weight gain. Oxygen in the chamber was regulated by using a ProOx110 controller (BioSpherix, US). Pups from each litter were randomly distributed at each experimental point. The n number given for each experiment describes the number of mice placed at the same time in the chamber but not necessarily the same litter. We measured the extent of retinal vascularization and pre-retinal neovascularization in retinal flat-mounts at the end of the period of hyperoxia, and subsequently at intervals for 14 days. We measured retinal VEGF and GFAP by qPCR, and investigated the impact of each protocol on retinal function by electroretinography. While the litters were exposed to hyperoxia, their nursing mothers were rested in room air for 2 hours daily. We observed the mothers’ health and investigated hyperoxia-induced lung injury by histological examination.

### Histology

Eyes were enucleated and fixed by immersion in 4% paraformaldehyde for 1 hour. Retinas were isolated by dissection, and flat mounted following incubation with biotinylated isolectin B4 (Sigma-Aldrich, 1/200 from 1mg/ml stock) and Alexa Fluor 546 conjugated streptavidin (Life Technologies). Images were taken by using a 10x objective and tilescan options. The extent of retinal vascular regression (central avascular area) and pre-retinal vascularization (NV) were measured using Image J [5], by quantification of manually selected area and intensity threshold- selected particles, respectively. For GFAP staining, eyes were fixed in 1% paraformaldehyde for 1 hour, embedded in OCT (RA Lamb) and frozen in isopentane precooled in liquid nitrogen. Sections of 18 μm of thickness were incubated with rabbit anti-GFAP antibody (Dako) and anti-rabbit-488 secondary antibody (Life Technologies). Lungs of the nursing mothers were dissected and fixed by immersion in 4% paraformaldehyde for 12 hours. DeadEnd^™^ Fluorometric TUNEL System kit (Promega) was used to determine apoptosis in 10, non-consecutive lung cryosections. Alternatively, cryosections were incubated with anti-rat CD45 (Millipore) or Van Gieson stain.

### Quantitative PCR

RNA was extracted from isolated retinas using the RNeasy mini kit (Qiagen) and cDNA produced using the QuantiTect Reverse Transcriptase kit (Quiagen). qPCR was performed with specific oligos for each gene using the TaqMan probe-based PerfeCTa^®^ qPCR FastMix^®^ (VWR) in an Applied Biosciences 7900HT thermocycler (Life Technologies).

### Electroretinography

Electroretinography was performed under scotopic and photopic conditions at P26 and P60. ERGs were obtained using an Espion E2 ERG system (Diagnosys). Multiple white-light flash intensity series were performed, with light intensity increasing in 10 steps (from 0.000001 to 75.20 cd.s/m^2^) for scotopic examination and 6 steps (from 0.1 to 75.20 cd.s/m^2^) for photopic conditions. The a-wave was defined as the maximal negative amplitude after the onset of light exposure. Measurements for b-wave amplitude were taken from the trough of the a-wave to the peak of the b-wave.

### Health of nursing mothers

We graded evidence of distress in nursing mothers during hyperoxia exposure by scoring behavioral features: orbital tightening, nose bulge, cheek bulge, ear position and whisker change (0: not present, 1: moderate, 2: obvious).

### Statistical analysis

**Fig 1:**
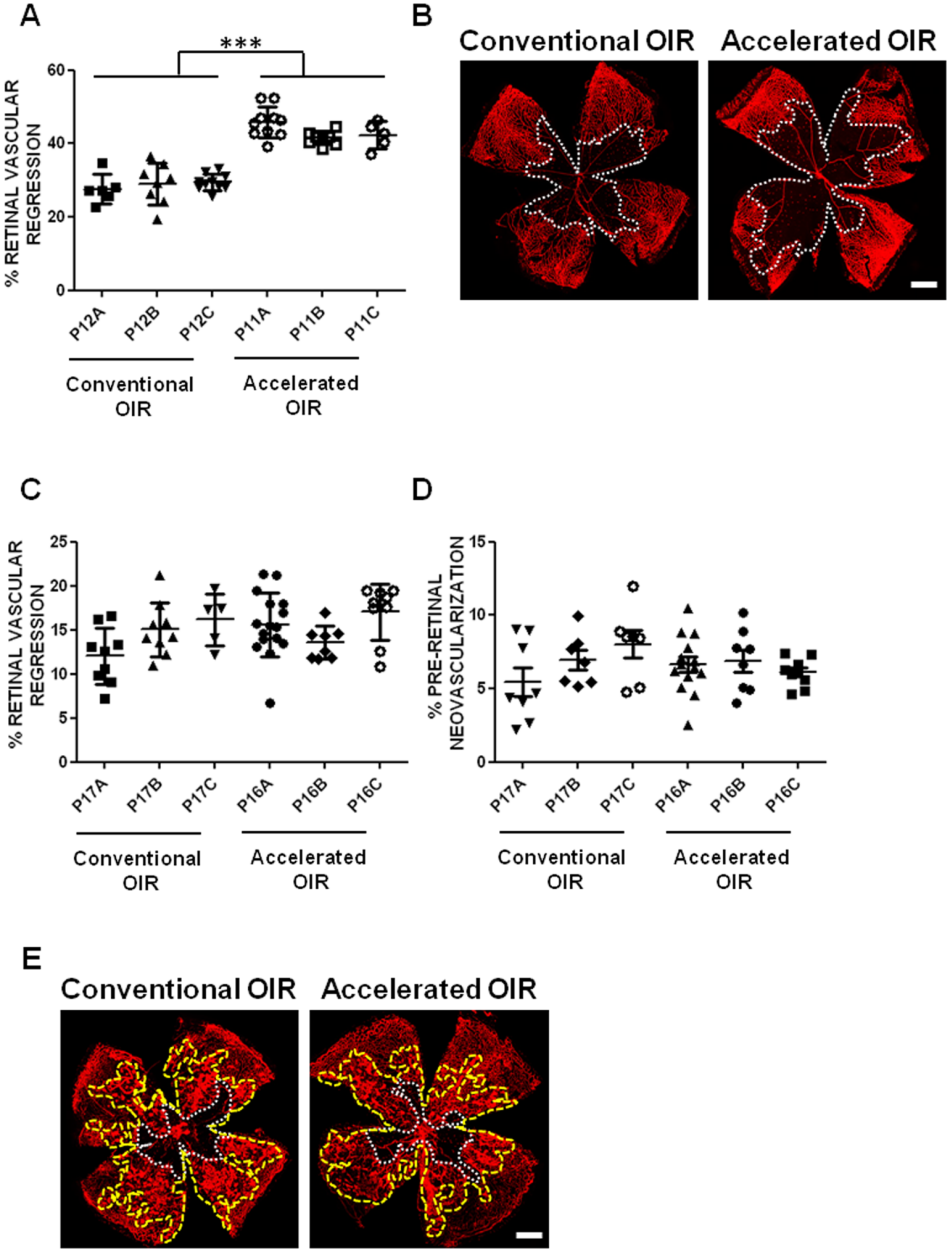
Retinal vasculature regression and pre-retinal neovascularization following hyperoxia and 5 days in room air. **(A)** The extent of oxygen-induced retinal vascular regression was consistently greater (in 3 independent experimental groups) in the accelerated protocol (85% O_2_ from P8 to P11) than in the conventional protocol (75% O_2_ from P7 to P12). No significant differences were evident between independent experimental groups within each protocol, and the variances were similar (Barlett's test, p = 0.6429 for retinal vascular regression and p = 0.1415 for pre-retinal neovascularization). **(B)** Representative images show the extent of retinal vascular regression (delineated in white) in isolectin B4-stained flat-mounted retinas of mice in the conventional and the accelerated OIR protocols. Five days following return to room air, the extents of both persistent retinal vascular regression **(C)** and pre-retinal neovascularization **(D)** were similar in retinas of mice in both protocols. **(E)** Representative images of flat-mounted retinas of mice from the conventional and accelerated protocols illustrate the area of persistent retinal vascular regression (delineated in white), and the area of pre-retinal neovascularization (delineated in yellow). Scale bars: 0.5 mm. n = 6–14 per group. Data are expressed as means ± SEM.

**Fig 2:**
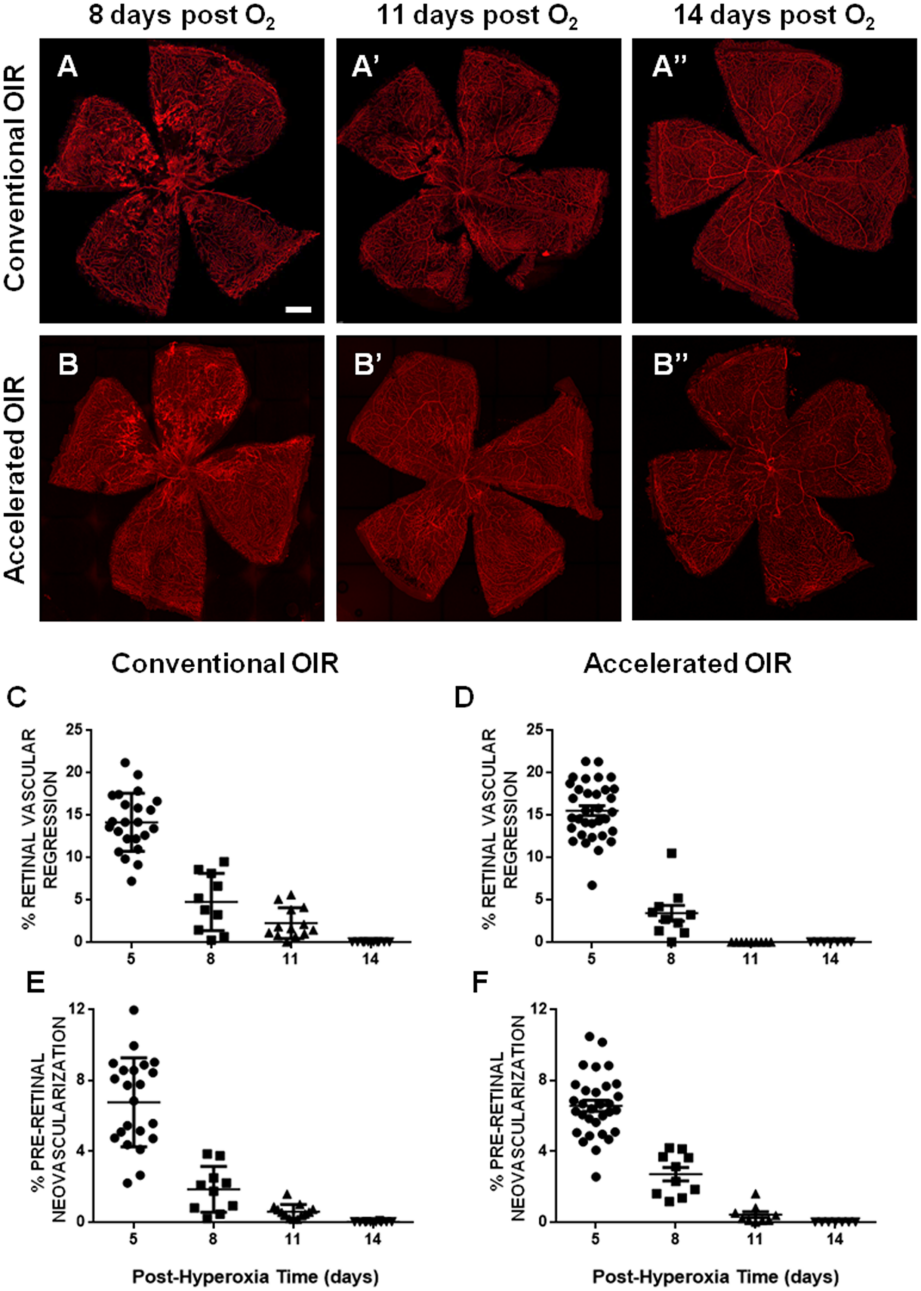
Time courses of retinal vasculature regeneration and pre-retinal neovascular regression. **(A, B)** Representative flat-mounted retinas from mice exposed to conventional OIR (75% O_2_, upper panel) or accelerated OIR (85% O_2_, lower panel) at 8 **(A, B)**, 11 **(A’, B’)** and 14 days after the end of hyperoxia **(A”,B”)**, showing progressive retinal vascular regeneration, and regression of the neovascular tufts. **(C, D)** Analysis of the persistent retinal vascular regression demonstrated more rapid vascular regeneration in the accelerated protocol at 11 days post-hyperoxia (p = 0.015). **(E, F)** No differences were found in the kinetics of regression of neovascular lesions between the two experimental conditions. Scale bars: 0.4 mm. n = 7–13 per group. Data are expressed as means ± SEM.

**Fig 3:**
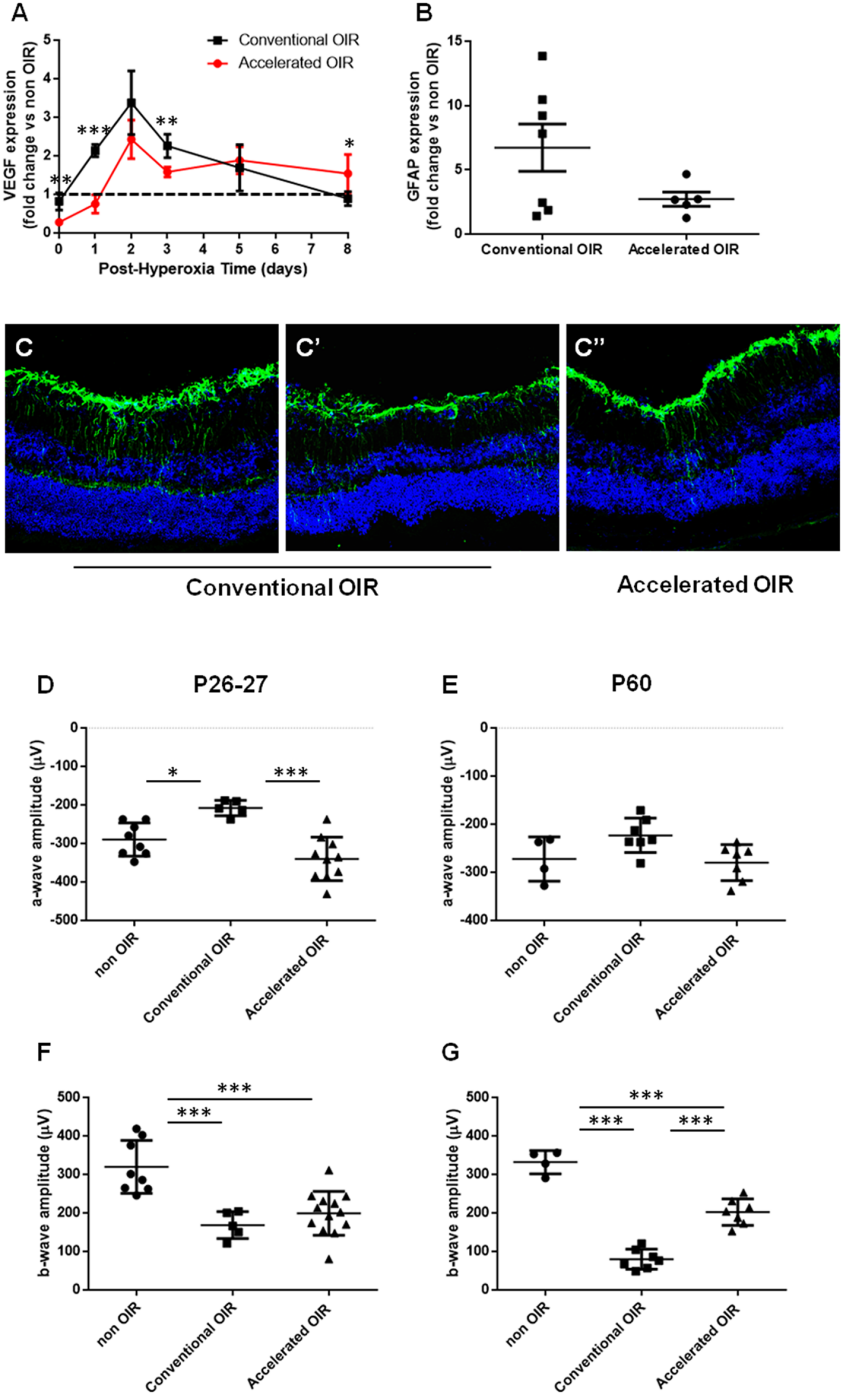
Molecular features and neuroretinal function. **(A)** Retinal VEGF was measured by qPCR at intervals (0,1,2,3,5 and 8 days) following exposure to hyperoxia (n = 4–7 per group. **(B)** GFAP expression measured by qPCR (n = 5–7 per group). **(C)** Representative images of GFAP immunostaining in retinal sections from mice after the conventional and the accelerated protocol. **(C)** and **(C’)** show the variability within the conventional OIR group. **(D to G)** Electroretinographic (ERG) a-wave and b-wave amplitudes in scotopic conditions were measured at P26-27 **(D, E)** and at P60 **(F, G)** in mice previously exposed to the conventional and accelerated OIR protocols, and in age-matched non-OIR controls (n = 4–13 per group). Data are expressed as means ± SEM.

**Fig 4:**
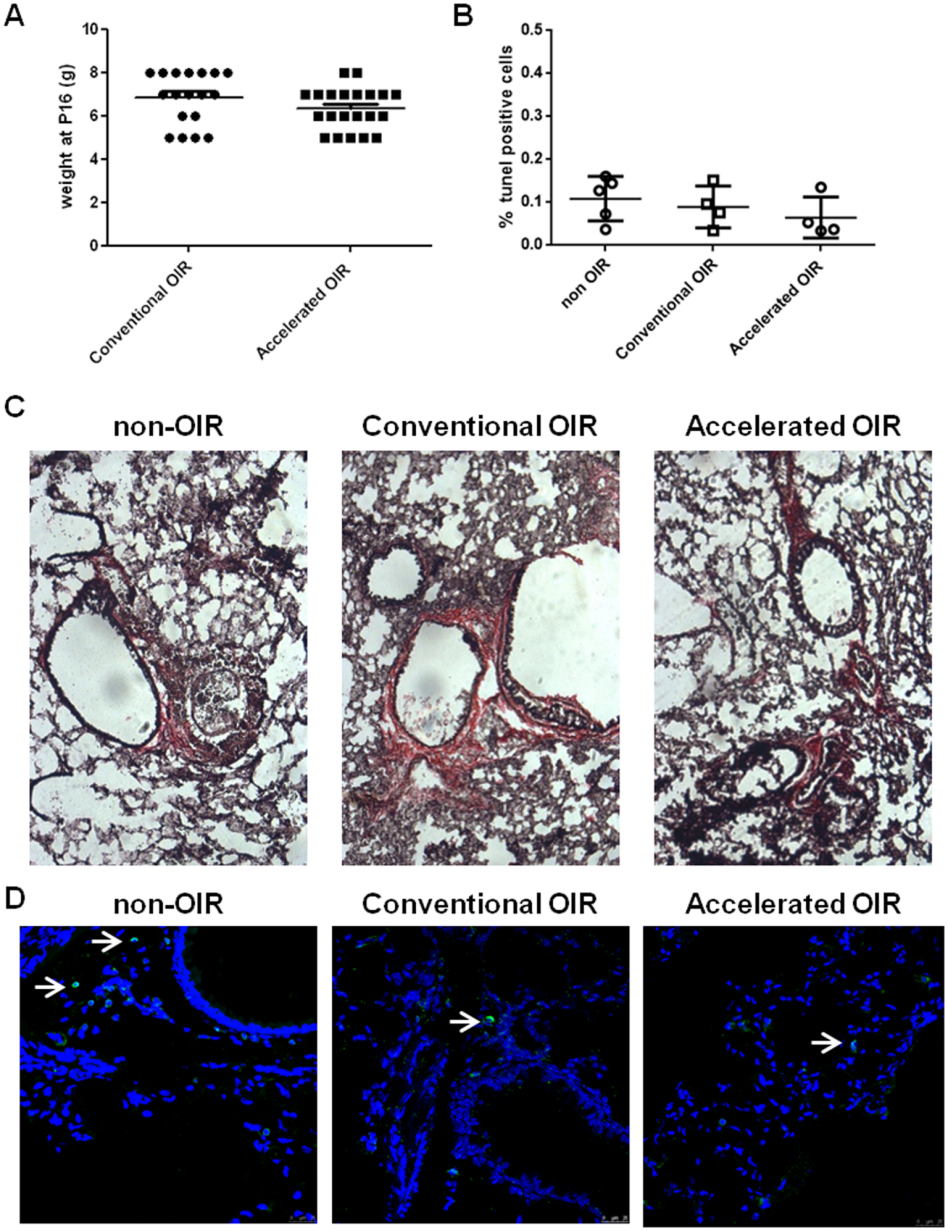
Histological analysis of lungs from nursing mothers after exposure to hyperoxia. **(A)** Body weight of pups at P16. **(B)** Alveolar cell apoptosis indicated by TUNEL positive cells in lung cryosections from non-OIR control mice and mice after conventional or accelerated OIR. **(C)** Van Gieson staining for collagen in lung cryosections shows no fibrotic lesions in any of the groups analyzed. Original magnification: 20x. **(D)** CD45-staining of cryosections demonstrates few CD45-positive cells in the lungs of non-OIR control animals and those after conventional or accelerated OIR.

Data was analyzed by using GraphPad Prism (Graphpad Software Inc.). N = number of eyes examined (Figs 1–3, one eye per animal was used for any given method of assessment) and n = number of lungs examined (Fig 4, one lung per animal was used for any given method of assessment). We compared 2 unmatched groups by unpaired Student’s t-tests, and 3 or more unmatched groups by one-way ANOVA with the Bonferroni correction for multiple comparisons. We compared variances between experimental groups by Bartlett tests. ***p<0.001, **p<0.01, *p<0.05.

## Results

### Accelerated OIR induces similar pre-retinal neovascularization but greater retinal vascular regression

Hyperoxia exposure during retinal vascular development induces regression of the developing retinal vasculature [1]. On return to room air, the resulting local ischemia and tissue hypoxia induce both appropriate regeneration of the retinal vasculature and aberrant growth of neovascular tufts that extend pre-retinally from the surface of the inner retina into the vitreous before regressing spontaneously. To compare the effect of the accelerated protocol with that of the conventional protocol we measured, following hyperoxia, the extents of retinal vascular regression and, after 5 days, the extents of retinal vascular regeneration and preretinal neovascularization. We found that accelerated OIR resulted in 40% greater retinal vascular regression (Fig 1A and 1B) following the period of hyperoxia, than the conventional model. Retinal vascular regeneration during the subsequent 5-day period in room air was also greater in the accelerated model, such that after 5 days the extent of persistent retinal vascular regression was similar to that in the conventional model (Fig 1C). The extent of pre-retinal neovascularization after 5 days in room air was similar to that in the conventional model (Fig 1D and 1E).

In OIR, both ischaemia-induced development of pre-retinal revascularization and its subsequent spontaneous regression occur within 14 days of hyperoxia [1, 2]. To investigate the time-courses of these processes in the 2 protocols we examined retinas at intervals following return to room air. After 11 days in room air, the extent of retinal vascular regeneration was greater in the accelerated model than in the conventional model (p = 0.015). Retinal vascular regeneration was complete in both models within 14 days of exposure to room air but this was achieved earlier in the accelerated model than in the conventional model (Fig 2A” and 2B”). Regression of pre-retinal neovascularization in the accelerated model followed a similar time-course to that of the conventional model.

### Accelerated OIR induces greater downregulation of retinal VEGF expression

Normal retinal vascular development is controlled by local expression of vascular endothelial growth factor (VEGF) [6]. In OIR, hyperoxia induces regression of the developing retinal vasculature by a mechanism involving local suppression of VEGF expression [7] and/or oxidative injury to endothelial cells [8, 9]. We measured total retinal VEGF expression by qPCR at intervals following exposure to hyperoxia in the conventional and accelerated models. The accelerated protocol (85% O_2_ from P8 to P11) resulted in greater suppression (to 30% of age-matched non-OIR controls) of retinal VEGF expression than the conventional protocol (to 80% of the non-OIR control) (p = 0.0013) (Fig 3A). Following return to room air, retinal VEGF expression was upregulated in both models reaching a similar peak after 2 days. Subsequently, retinal VEGF expression declined, returning to normal levels in both protocols within 8 days, though the rate of decline appeared slower in the accelerated model than in the conventional model (p = 0.024). Retinal GFAP expression, which is a hallmark of retinal gliosis, was upregulated in astrocytes and Muller cell bodies in both OIR protocols. The extent of GFAP upregulation was highly variable in the conventional OIR model (Fig 3B and 3C) and appeared less marked in the accelerated model, though the difference in this experiment was not statistically significant (p = 0.11).

### Accelerated OIR causes less severe neuroretinal dysfunction

Since exposure to hyperoxia can cause neuroretinal dysfunction [10] we compared the impact of the accelerated and conventional protocols on neuroretinal function at P26-27 (Fig 3D and 3F) and at P60 (Fig 3E and 3G) by measuring the amplitudes of electro responses to light stimuli in scotopic (dark-adapted, rod-mediated) conditions. The conventional protocol resulted in a reduction in a-wave amplitude at P26-27 and a marked sustained reduction in b-wave amplitude. The accelerated protocol caused no impact on a-wave amplitude; a sustained reduction in b-wave amplitude was evident but this was less marked than that resulting from the conventional model. Photopic, cone-mediated responses were similarly affected (S1 Fig).

### Safety

Exposure to hyperoxia is generally well tolerated by mouse pups; we observed no mortality and their mean weight at P16 was similar in both protocols (Fig 4A). However, OIR can cause hyperoxia-induced respiratory distress in nursing mothers, especially when exposed to concentrations higher than 80% (1). To compare the impact of the accelerated model with that of the conventional model we observed nursing mothers (n = 23) for signs of distress on their return to room air and 5 days later. We observed no signs of maternal distress or respiratory problems and no maternal mortality associated with either of the protocols. Histological examination of the lungs of nursing mothers 5 days after the end of hyperoxia demonstrated no increased inflammation (Fig 4B), fibrosis (Fig 4C) or alveolar cell apoptosis (Fig 4D) to indicate hyperoxic lung injury [11].

## Discussion

Direct comparison with the conventional model of OIR identifies features of the accelerated model that offer distinct advantages. The accelerated OIR protocol safely and reliably induces similar pre-retinal neovascularization despite a shorter duration of hyperoxia, offering a more refined and efficient experimental protocol. The accelerated protocol results in more profound hyperoxia-induced downregulation of VEGF and more extensive retinal vascular regression, offering a greater opportunity to investigate endogenous mechanisms and potential interventions that promote vascular regeneration.

OIR is associated with retinal macroglial cell activation and neuroretinal dysfunction [12]. Retinal GFAP overexpression, which is evident in a broad range of pathological conditions [12, 13], appeared milder in the accelerated model, though this was highly variable in the conventional model and the difference not statistically significant. The accelerated model, however, is less vulnerable to hyperoxia-induced neuroretinal dysfunction. In OIR, hypoxia/ischemia of the inner retina leads to inner retinal dysfunction evident on electroretinography as reduced b-wave amplitude [10]. This effect was less marked in the accelerated version, suggesting that any adverse impact of the greater hyperoxia is mitigated by its briefer duration. Better preservation of the a-wave at the earlier time-point indicates that that the timing and level of hyperoxia is also better tolerated by photoreceptor cells, which are vulnerable to direct hyperoxic oxidative stress [10]. The differential vulnerability to hyperoxia-induced neuroretinal dysfunction highlights opportunities to investigate the relevant pathogenic mechansims and potential interventions.

The accelerated OIR protocol offers a refined alternative model that is safe, reliable and efficient; accelerated OIR induces similar pre-retinal neovascularization but greater retinal vascular regression that recovers more rapidly and neuroretinal function is better preserved.

## Supporting information

S1 Fig Photopic ERG responses after the conventional and the accelerated OIR protocol. **(A)** Only the conventional OIR induced the reduction of the a-wave amplitudes at P27. Responses were normalized at P60 **(B)**. **(C)** B-wave amplitudes showed a greater reduction at P26-27 after the conventional OIR compared with the accelerated version. Responses were normalized at P60 **(D)**. n = 4–13 per group. Data are expressed as means ± SEM.
(TIF)

S1 Checklist NC3Rs ARRIVE guidelines checklist. (PDF)
