# Supplementary figures and images for: Accelerated oxygen-induced retinopathy is a reliable model of ischemia-induced retinal neovascularization
Source: PLoS One. 2017 Jun 26;12(6):e0179759. doi: 10.1371/journal.pone.0179759 (PMC5484470; doi:10.1371/journal.pone.0179759)

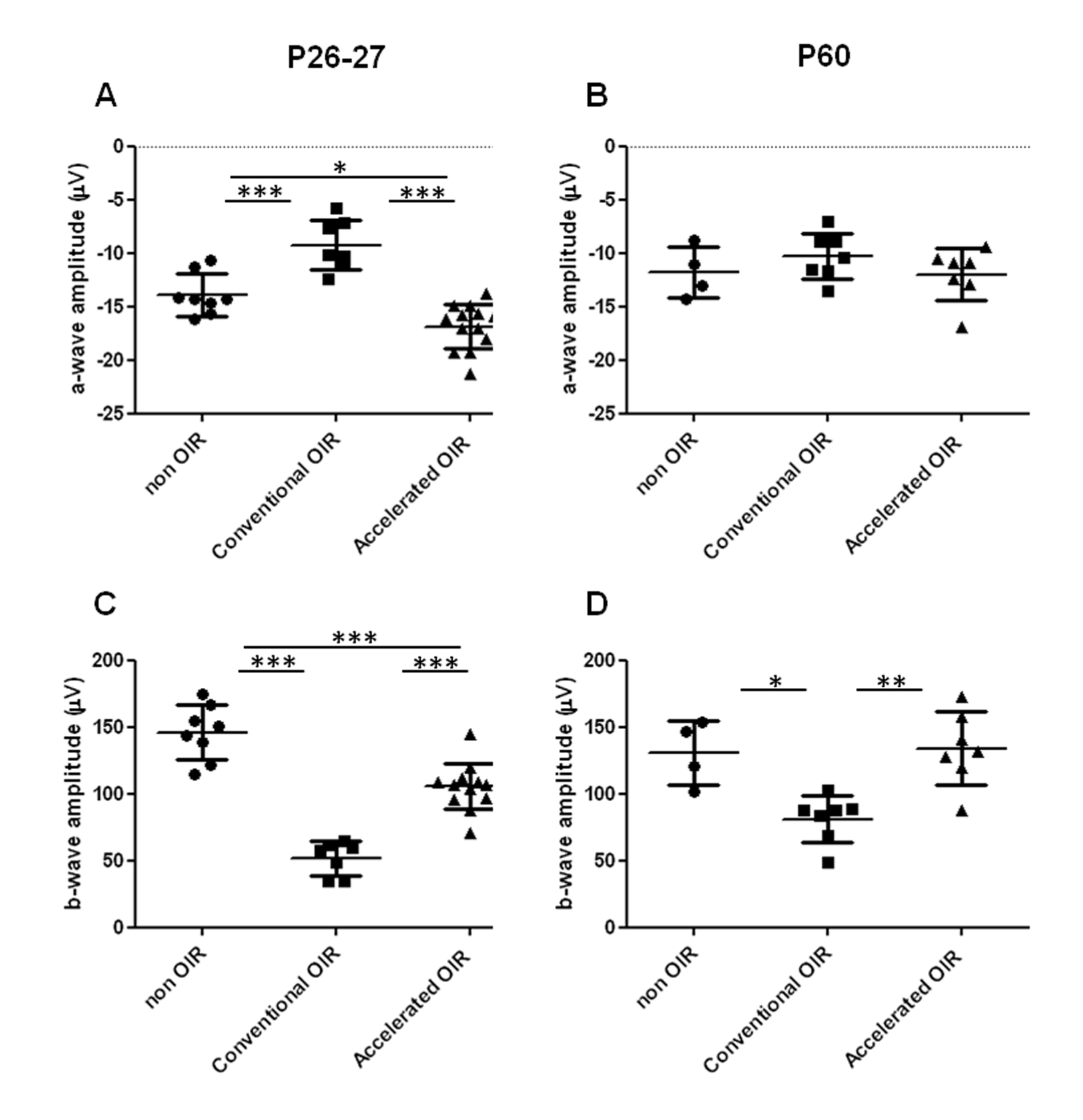

Supplement: S1 Fig — (A) Only the conventional OIR induced the reduction of the a-wave amplitudes at P27. Responses were normalized at P60 (B). (C) B-wave amplitudes showed a greater reduction at P26-27 after the conventional OIR compared with the accelerated version. Responses were normalized at P60 (D). n = 4–13 per group. Data are expressed as means ± SEM. (TIF) [file pone.0179759.s001.tif]
